# β2-adrenergic signal transduction plays a detrimental role in subchondral bone loss of temporomandibular joint in osteoarthritis

**DOI:** 10.1038/srep12593

**Published:** 2015-07-29

**Authors:** Kai Jiao, Li-Na Niu, Qi-hong Li, Gao-tong Ren, Chang-ming Zhao, Yun-dong Liu, Franklin R. Tay, Mei-qing Wang

**Affiliations:** 1 State Key Laboratory of Military Stomatology, Department of Oral Anatomy and Physiology and TMD, School of Stomatology, Fourth Military Medical University, 145 Changle Western Road, Xi’an, China 710032; 2 State Key Laboratory of Military Stomatology, Department of Prosthodontics, School of Stomatology, Fourth Military Medical University, Changle Western Road No.145, Xi’an, China 710032; 3 Department of Stomatology, Affiliated Hospital of Academy of Military Medical Science, Beijing, China; 4 Undergraduate Department of Oral Science, Fourth Military Medical University, Changle Western Road No.145, Xi’an, China 710032; 5 College of Dental Medicine, Georgia Reagents University, Augusta, GA, USA

## Abstract

The present study tested whether activation of the sympathetic tone by aberrant joint loading elicits abnormal subchondral bone remodeling in temporomandibular joint (TMJ) osteoarthritis. Abnormal dental occlusion was created in experimental rats, which were then intraperitoneally injected by saline, propranolol or isoproterenol. The norepinephrine contents, distribution of sympathetic nerve fibers, expression of β-adrenergic receptors (β-ARs) and remodeling parameters in the condylar subchondral bone were investigated. Mesenchymal stem cells (MSCs) from condylar subchondral bones were harvested for comparison of their β-ARs, pro-osteoclastic gene expressions and pro-osteoclastic function. Increases in norepinephrine level, sympathetic nerve fiber distribution and β2-AR expression were observed in the condylar subchondral bone of experimental rats, together with subchondral bone loss and increased osteoclast activity. β-antagonist (propranolol) suppressed subchondral bone loss and osteoclast hyperfunction while β-agonist (isoproterenol) exacerbated those responses. MSCs from experimental condylar subchondral bone expressed higher levels of β2-AR and RANKL; norepinephrine stimulation further increased their RANKL expression and pro-osteoclastic function. These effects were blocked by inhibition of β2-AR or the PKA pathway. RANKL expression by MSCs decreased after propranolol administration and increased after isoproterenol administration. It is concluded that β2-AR signal-mediated subchondral bone loss in TMJ osteoarthritisis associated with increased RANKL secretion by MSCs.

Osteoarthritis (OA), the most common degenerative joint disorder, is characterized by progressive cartilage degradation and subchondral bone changes^1^. Approximately 27 million adults in the US suffered from OA in 2005^2^, and the prevalence is expected to increase to 67 million by 2030^3^. Abnormal subchondral bone remodeling plays an important role in the pathogenesis of OA^4^. Reduced bone mineral density and increased subchondral bone remodeling have been observed in the early stages of OA^5^,^6^. Loss of subchondral bone further triggers degradation of the overlying cartilage through aggravation of the biomechanical environment^7^. Bone resorption inhibitors such as pamidronate disodium^8^, bisphosphonates^9^, strontium ranelate^10^ and osteoprotegerin^11^ have been shown to reduce pathological features associated with OA in experimental animal models. These results highlight the necessity for better understanding of the biological factors involved in pathological remodeling of subchondral bone in OA.

Abnormal mechanical loading has been considered the most important pathogenic factors in the development and progression of OA^12^. Alteration of the loading environment in knee joints of rodents through transection of the anterior cruciate ligament^13^, destabilization of the meniscus^14^ or cyclic compression of the joint^15^, induced progressive subchondral trabecular bone loss during the early post-operative period. The temporomandibular joint (TMJ) is one of the most common sites of OA^16^. Osteoarthritis-like lesions were observed when aberrant mechanical loading was induced in rodent TMJ through abnormal dental occlusion in posterior-teeth^17^,^18^,^19^ or anterior-teeth^20^,^21^,^22^. Despite meticulous documentations of these cause-effect relationships, the etiopathogenic mechanism of condylar subchondral bone loss induced by aberrant mechanical loading remains obscure.

Mammalian bones are profusely innervated by sympathetic nerves^23^. During normal bone remodeling, norepinephrine release by sympathetic nerves suppresses bone formation and promotes bone resorption. Such a process is mediated by the β-adrenergic receptors (β-ARs) expressed by osteoblasts and osteoclasts. There are 3 receptor subtypes in the β-AR family: β1, β2 and β3. Stimulation of β2-AR (Adrb2) inhibits proliferation and differentiation of osteoblasts, but promotes maturation and bone-resorbing activity of osteoclast precursors^24^. Recent studies have shown that the sympathetic nerve system is involved in regulating bone mechanoadaptive responses. Blocking of sympathetic signals by chemical sympathectomy or the use of a non-selective β-blocker (propranolol) prevented loading-induced hind limb bone loss in rodent models^25^,^26^. Elimination of sympathetic signals by surgical sympathectomy, the use of a selective Adrb2 antagonist (butoxamine), or Adrb1/2 globe knockout suppressed alveolar bone loss and osteoclast hyperactivities induced by occlusal hypofunction^27^ or experimental orthodontic forces^28^,^29^,^30^. Although sprouting of sympathetic nerve fibers has been identified from subchondral bone in osteoarthritic knee joints^31^,^32^, it is not known whether the sympathetic tone is responsible for regulating abnormal subchondral bone remodeling in OA.

Mesenchymal stem cells (MSCs) derived from bone marrow is the progenitor cell of osteoblasts. They secret cytokines such as receptor activator of nuclear factor kappa-β ligand (RANKL) and osteoprotegerin (OPG) to modulate the development of osteoclasts from their precursors. Hence MSCs play important roles in bone remodeling^33^,^34^,^35^. Recent studies have shown that MSCs are innervated by adrenergic nerve fibers of the sympathetic nervous system^36^,^37^. Activation of β2-AR signal in MSCs suppresses their osteogenic differentiation potential^38^, and modulates their chemokine expression for regulating the homeostasis of hematopoietic stem cells^39^. Although osteoclasts originate from hematopoietic stem cells, it is not known whether activation of sympathetic receptors of MSCs affect the development of osteoclasts.

The present study was conducted to bridge these knowledge gaps by testing the hypothesis that activation of the sympathetic tone induced by aberrant joint loading elicits osteoclast-mediated subchondral bone remodeling in a rat TMJOA model. To address this hypothesis, abnormal dental occlusion was created in experimental rats to examine whether abnormal occlusion activates sympathetic tone in condylar subchondral bone, and whether inhibition of β-adrenergic signals prevents condylar subchondral bone loss. The pro-osteoclastic effects induced by norepinephrine stimulation of MSCs were further examined to understand the underlying mechanism responsible for norepinephrine-induced bone resorption.

## Materials and Methods

### Animal model

All animal procedures were performed according to the guidelines of the Animal Care Committee of the Fourth Military Medical University, Xi’an, China, and all experimental protocols were approved by Fourth Military Medical University. Six-week old female Sprague-Dawley rats (180–190 g) were obtained from the Animal Center of Fourth Military Medical University. In the experimental groups, a unilateral anterior crossbite prosthesis was bonded to the lower incisors of each rat to induce abnormal mechanical loading on its TMJs^21^,^22^. Rats in the control groups underwent a mock operation procedure without permanent bonding of the unilateral anterior crossbite prosthesis. For pharmacological treatment, each rat received intraperitoneal injections of physiological saline (Veh), a non-selective β-blocker (propranolol; 20 μg/g, PRO, Sigma-Aldrich, St. Louis, MN, USA), or a β-adrenergic receptor agonist (isoproterenol; 5 μg/g, ISO, Sigma-Aldrich)^26^,^28^. The rats were treated twice a week from the first day when the dental occlusion was disturbed. They all received the same standardized diet and no rat showed any signs of disability during the experimental period.

### Group designation and sampling

Rats from the experimental and control groups were sacrificed at the end of 2, 4 or 8 weeks. Sera derived from 6 rats in each group (2 × 3 time-periods = 36 rats) were collected for measurement of serum norepinephrine concentrations. Because no differences in degrading changes were identified between the left side and right side of the TMJs in the experimental rats in our previous reports^21^,^22^, both condyles of these 6 rats were used for the detection of norepinephrine levels in condylar subchondral bone. The left condylar tissue blocks were harvested from six additional 4-week experimental and control rats, fixed, decalcified and embedded in paraffin for preparation of 5 μm-thick sagittal sections. The sections were immunohistochemically-stained for detection of sympathetic nerve fibers (as demonstrated by positive staining for tyrosine hydroxylase, the rate-limiting enzyme of norepinephrine biosynthesis)^28^, and Adrb2-positive cells in the condylar subchondral bone. The right condylar subchondral bones were harvested for real time-polymerase chain reaction (RT-PCR). For pharmacological manipulation, the 4-week and 8-week experimental rats were further divided into 3 subgroups: Exp + Veh, Exp + PRO and Exp + ISO (N = 6). The left condyles were used for micro-computed tomography (GE eXplore Locus SP, London, United Kingdom), and the right condylar tissue blocks were fixed, decalcified, paraffin-embedded and sectioned. Five micrometer-thick sagittal sections were used for hematoxylin-and-eosin (HE), tartrate-resistant acid phosphatase (TRAP) and osteocalc inimmunohistochemical staining.

### Norepinephrine determination

Norepinephrine contents in the serum and condylar subchondral bone were measured using enzyme-linked immunosorbent assay (BA E-5200, Rocky Mountain Diagnostics, Colorado Springs, CO; USA)^40^. Briefly, condylar subchondral bone from 6 individual rats were pooled, weighed and homogenized in 0.01 N HCl to avoid association of catecholamines with other proteins. Blood sample from each rat was centrifuged at 3,000 rpm for 10 min immediately after collection; the serum was frozen until analysis. Norepinephrine was extracted using a cis-diol specific affinity gel, acylated and enzymatically-converted to *N*-acylmetanephrine. The *N*-acylmetanephrine in the sample competed with *N*-acylmetanephrine immobilized on the microtiter plate for binding to a specific antibody. Following rinsing to remove free *N-*acylmetanephrine-antibody complexes, the antibody bound to plate was detected by an anti-rabbit IgG-peroxidase conjugate using 3,3′,5,5′-tetramethyl benzidine as substrate. The final reaction products of norepinephrine were determined by measuring the optical density at 450 nm using a 96-well plate reader.

### Micro-computed tomography

Trabecular microstructure and bone mineral density (BMD) of condylar subchondral bone was analyzed by micro-computed tomography as previously described^17^,^18^. Briefly, two cubes (each 0.5 × 0.5 × 0.5 mm) were selected from the middle of the center and posterior thirds of condylar subchondral bone. Within the selected regions, BMD, bone volume fraction (BVF), bone surfacetovolumeratio (BS/BV), trabecular thickness(Tb.Th), trabecular number (Tb.N) and trabecular separation (Tb.Sp) were determined using the MicroView Advanced Bone Analysis 2.1.2 software (GE Healthcare, Pittsburgh, PA, USA).

### Bone histomorphometry

Hematoxylin-and-eosin and TRAP staining were performed as previously described^17^,^18^. Two square frames (each 0.5 × 0.5 mm) under the osteochondral interface were located at the middle of the center and posterior thirds of the mandibular condyle. Within the selected frames, BVF, Tb.Th, Tb.N, Tb.Sp, osteoclast number (Oc.N) and the percentage of osteoclast surface area with respect to bone surface (Oc.S/BS) were measured^17^.

### Immunohistochemical staining

Tissue processing, section staining and counting of immune-positive cells were performed as reported previously^17^,^18^,^19^. The primary antibodies were rabbit monoclonal tyrosine hydroxylase (TH; 1:100; ab75875, Abcam, Cambridge, MA, USA), rabbit polyclonal β2-adrenergic receptor (Adrb2; 1:100, ab137494; Abcam) and osteocalcin (1:100, sc-30045, Santa Cruz Biotechnology, Inc., Dallas, Texas, USA). Two square frames (each 0.5 × 0.5 mm) under the osteochondral interface were located at the middle of the center and posterior thirds of the mandibular condyle. Within the selected frames, the percentage area of TH-positive sympathetic nerve fibers, the number of Adrb2-positive cells and osteocalcin-positive osteoblasts were counted.

### MSCs isolation from TMJ and norepinephrine stimulation

Mesenchymal stem cells (MSCs) from the condylar subchondral bone of 4-week experimental and control rats were isolated and cultured as previously reported^41^. Briefly, all nucleated cells (ANCs) from condylar subchondral bones were obtained by digestion with 3 mg/mL collagenase type I and 4 mg/mL dispase II (Roche Diagnostic, Indianapolis, IN, USA). Single-cell suspensions of ANCs were obtained and seeded at 1.5 × 10^6^ on a 100-mm dish. The cells that formed single colonies were collected and passed to P1 expansion for subsequent experiments. Harvested MSCs (P1) were stained for cell-surface markers and analyzed by flow cytometry. Their multi-differentiation abilities were test in the osteogenic, adipogenic and chondrogenic culturing condition. The MSCs were treated with norepinephrine (N5785, Sigma-Aldrich) for 2, 6 and 24 h at 0.1, 1 and 10 μM, respectively. For inhibition assay, MSCs were treated with 1 μM norepinephrine for 2 h alone, or pre-treated for 1 h with 10 μM propranolol (P0884, Sigma-Aldrich), a non-selective β-adenoreceptor antagonist, 1 μM ICI 118,551 (0821, R&D Systems, Minneapolis, MN, USA), a specific β2-adenoreceptor antagonist, 5 μM H-89 (9844, Cell Signaling Technology, Inc., Danvers, MA,USA), a selective PKA inhibitor, or 5 μM U-0126 (9903, Cell Signaling Technology, Inc.), a selective ERK1/2 inhibitor. The inhibitor pre-treated MSCs were subsequently stimulated with 1 μM norepinephrine for 2 h.

### Culture of bone marrow macrophages (BMMs)

Bone marrow macrophages were obtained from the tibiae and femora of 8 week-old rats according to methods described previously^42^,^43^. Briefly, the bone marrow suspension was harvested by flushing the marrow space of femora and tibiae. The flushed bone marrow cells were cultured overnight on 100-mm culture dishes in α-MEM containing 10% FBS, 100 U/mL penicillin, 100 μg/mL streptomycin sulfate and 25 ng/mL macrophage colony-stimulating factor (M-CSF; R&D Systems). After discarding the adherent cells, floating cells were incubated with M-CSF (25 ng/mL) to obtain pure BMMs. After 3 days, adherent cells were used as BMMs for subsequent studies.

### *In vitro* osteoclastogenesis

Mesenchymal stem cells harvested from condylar subchondral bone were pre-seeded at a density of 1 × 10^4^ on 96-well Transwells (0.4 μm, Corning Life Science, Tewksbury, MA, USA) for 24 h. The MSCs were then stimulated for 2 h with 1 μM norepinephrine alone, or pre-treated respectively for 1 h with 10 μM propranolol, 1 μM ICI 118,551, 5 μM H-89, 5 μM U-0126 prior to stimulation with norepinephrine for 2 h. Thereafter, the seeded MSCs were co-cultured with 1 × 10^4^ BMMs pre-seeded onto the 96-well culture plate 24 h ago. The co-culturing medium was Dulbecco’s modified Eagle’s medium, that was supplemented with 25 ng/mL M-CSF, 10 ng/mL RANKL (R&D Systems) and 10% fetal bovine serum. The co-culturing medium was replaced on days 2 and 5 of co-incubation. After 7 days of co-incubation, the cells in the 96-well plates were fixed with 4% phosphate buffered saline-buffered paraformaldehyde and stained with TRAP (387A, Sigma-Aldrich). The total number of TRAP-positive multinucleated cells (three or more nuclei per cell) from ten randomly-selected 400xfields was counted under a light microscope (N = 6).

### *In vitro* pit formation

A similar co-culture assay as described in the osteoclastogenesis experiment was employed, except that the BMMs were pre-seeded on the top of sterilized dentin slices derived from extracted bovine teeth (1-mm thick) in the 96-well culture plate. After 14 days of culture, the BMMs were removed from the dentin slices by sonication for 5 min. The dentin slices were stained with 1% toluidine blue (Sigma-Aldrich) for 5 min, washed with distilled water and observed under a light microscope^42^. The stained resorption pits were visualized as blue areas. The mean area of resorption from six randomly-selected 400x fields was analyzed using image analysis software (Image J, National Institute of Health, Bethesda, MD, USA).

### RT-PCR

Gene expression of β-adrenergic receptors and cytokines related to osteoclast development, such as RANKL, OPG, monocyte chemotactic protein-1 (MCP-1), M-CSF, interleukin-6 (IL-6) and tumor necrosis factor-α (TNF-α)^44^, were detected by RT-PCR as described previously^17^,^18^. Briefly, total RNA was extracted using Trizol (Thermo Fisher Scientific, Waltham, MA, USA). Primers for target genes were listed in Supplementary Table I. Gene expression was analyzed with the 7500 real-time PCR (Thermo Fisher Scientific), using glyceraldehyde 3-phosphate dehydrogenase (GAPDH) as the internal control. The amount of target cDNA relative to GAPDH was calculated using the formula 2^-ΔΔCT^. Results were calculated as the relative quantification compared to the control group, which was set at 1. Data were collected from 3 independent pooled samples.

### Western blotting

Total protein from each group (40 mg) was fractionated by SDS-PAGE and transferred onto a nitrocellulose membrane. The nitrocellulose membrane was blocked with 5% non-fat milk and incubated with primary antibodies against Adrb2 (1:500, ab137494, Abcam), OPG (1:300, 11383, Santa Cruz Biotechnology, Inc.), RANKL (1:300, 7628, Santa Cruz Biotechnology, Inc.) and β-actin (1:1000, 3700, Cell Signaling Technology). Signals were revealed by incubation with a horseradish peroxidase-conjugated secondary antibody (1:5000, Zhongshan Goldenbridge Biotechnology, China) and enhanced chemiluminescence detection^19^.

### Statistical analyses

The measurement procedures for bone histomorphometry and cell counting were performed in a blinded fashion by two independent observers (GTR and CMZ) using Photoshop CS 7.0 software (Adobe Systems Incorporated, San Jose, CA, USA). The inter-observer reliability was analyzed by calculating the Intraclass Correlation Coefficient (ICC) for the measurements^20^,^45^. There was a high level of agreement between the two observers (ICC = 0.933) and the mean value of the two measurements from the same sample was used for further statistical analysis. Data were expressed as means ± standard deviation for each group. Normality of data distribution was tested by Shapiro-Wilk test with 95% confidence and Levene’s test was used to assess homogeneity of variance. The assumptions of parametric tests were fulfilled and statistical significance among groups was evaluated by analysis of variance. Post-hoc comparison between groups was performed using the Tukey test. P-values less than 0.05 were considered statistically significant for all tests.

## Results

### Body weight

All rats are healthy and there were no significant changes in body weight in any of the groups during the course of the study (data not shown).

### Abnormal occlusion induced increased level of norepinephrine, TH-immunoreactivity and Adrb2 expression in condylar subchondral bone

**Figure 1:**
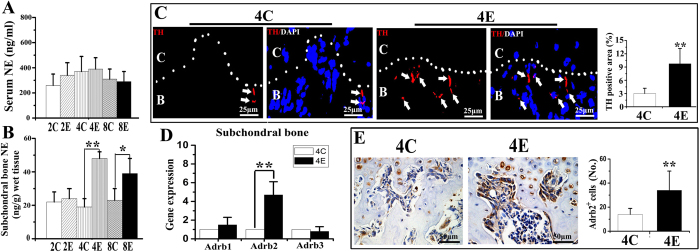
Detection of sympathetic tone in serum and condylar subchondral bone of 4-week control (4C) and 4-week experimental (4E) rats. **A, B**: Norepinephrine (NE) levels in serum (**A**) and condylar subchondral bone (**B**) over the three time-points (2, 4 and 8 weeks) examined. **C**: control rats; E: experimental rats. Levels of significance: *P < 0.05, **P < 0.01. **C**: Immunofluorescent staining and quantification of the tyrosine hydroxylase (TH) positive sympathetic nerve fibers in the condylar subchondral bone of 4-week control and experimental groups. The dash line indicates the interface between cartilage (**C**) and subchondral bone (**B**). Arrows indicate TH-positive sympathetic nerve fibers (red color). The blue color indicates cell nuclei stained by DAPI. **D**: Real-time PCR analysis of the mRNA expression of β-adrenergic receptors (β-ARs) in the condylar subchondral bone of 4-week control and experimental groups. **E**: Immunohistochemical staining and quantification of the β2-AR (Adrb2) positive cells in the condylar subchondral bone of 4-week control and experimental groups. Levels of significance for all charts: *P < 0.05, **P < 0.01.

Serum norepinephrine levels were not significantly different among experimental and control groups for all time periods (p > 0.05; Fig. 1A). The levels of norepinephrine in condylar subchondral bone were significantly increased in 4-week and 8-week experimental groups comparing to their age-matched controls (both p < 0.05; Fig. 1B), while that in 2-week experimental group was not significantly different from its age-matched control (p = 0.226; Fig. 1B). We then focus on 4-week groups for further observation.

In 4-week control rats, TH-positive sympathetic nerve fibers and Adrb2-positive cells were barely noticeable beneath the osteochondral interface (Fig. 1C,E). In contrast, robust sprouting of TH-positive sympathetic nerve fibers (Fig. 1C), and increased Adrb2 mRNA expression (Fig. 1D) and Adrb2 immuno-positive cells (Fig. 1E) were observed in the condylar subchondral bone of 4-week experimental rats (all p < 0.05). The mRNA expression of Adrb1 and Adrb3 did not exhibit any difference between the 4-week control and experimental groups (p > 0.05; Fig. 1D).

### Propranolol suppressed, while isoproterenol intensified subchondral bone loss and osteoclast hyperfunction induced by abnormal occlusion

**Figure 2:**
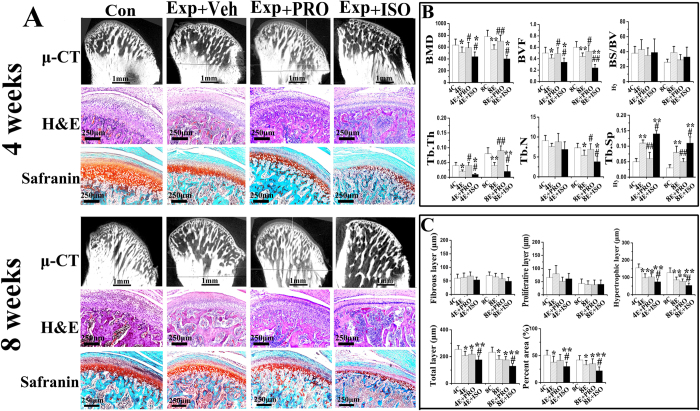
Condylar subchondral bone and cartilage changes of 4-week and 8-week control rats (Con) and experimental rats (Exp) treated with physiological saline (Veh), non-selective β-blocker propranolol (20 μg/g, PRO), or β-adrenergic receptors agonist isoproterenol (5 μg/g, ISO). **A**: Representative images of micro-computed tomography, H&E and safranin O staining of the mandibular condye. **B**: Analysis of bone mineral density (BMD) and different parameters representing trabecular microstructures of the condylar subchondral bone based on the reconstructed micro-computed tomography images. **C**: Comparison of the values of the thickness of fibrous, proliferative, hypertrophic, and total layers of the condylar cartilage and the percent area of safranin O staining. *P < 0.05: *vs* age-matched control, **P < 0.01: *vs* age-matched control; ^#^P < 0.05: *vs* age-matched vehicle-treated counterpart, ^##^P < 0.01: *vs* age-matched vehicle-treated counterpart.

**Figure 3:**
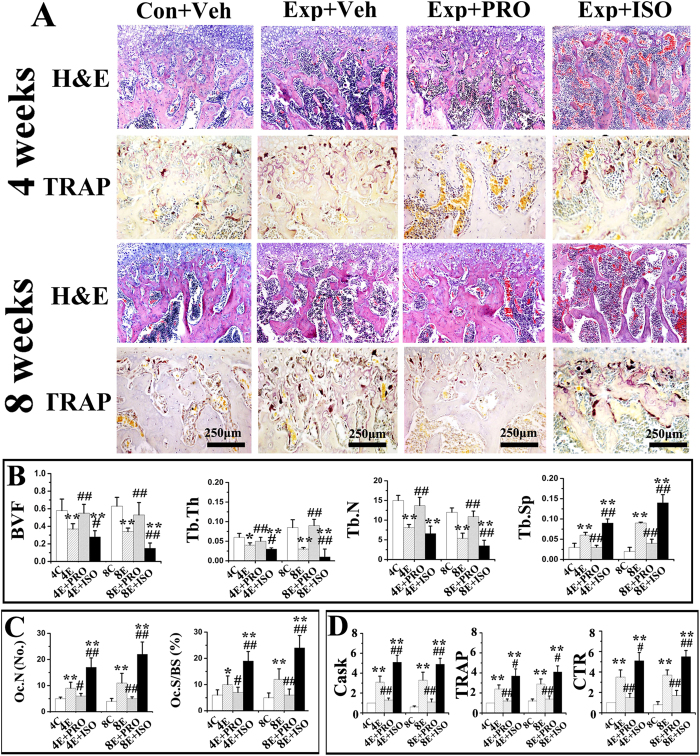
Bone histomorphometry and detection of osteoclastic activities of the condylar subchondral bone of 4-week and 8-week control rats (Con) and experimental rats (Exp) treated with physiological saline (Veh), propranolol (PRO) or isoproterenol (ISO). **A**: Representative images of H&E and TRAP staining. **B**: Bone histomorphometric analysis of bone volume fraction (BVF), trabecular thickness (Tb.Th), trabecular number (Tb.N) and trabecular separation (Tb.Sp). **C**: Bone histomorphometric analysis of osteoclast number (Oc.N) and the percentage of osteoclast surface area with respect to bone surface (Oc.S/BS). **D**: Real-time PCR analysis of the mRNA expression of Cathepsin K (CasK), TRAP and calcitonin receptor (CTR) in the condylar subchondral bone. *P < 0.05: *vs* age-matched control, **P < 0.01: *vs* age-matched control; ^#^P < 0.05: *vs* age-matched vehicle-treated counterpart, ^##^P < 0.01: *vs* age-matched vehicle-treated counterpart.

**Figure 4:**
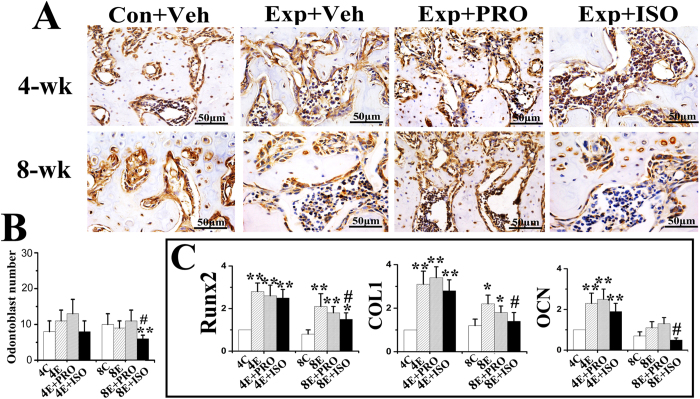
Osteocalcin immunohistochemical staining of condylar subchondral bone of 4-week and 8-week control rats (Con) and experimental rats (Exp) treated with physiological saline (Veh), propranolol (PRO) or isoproterenol (ISO). The number of osteocalcin-positive cells within the selected area was counted. *P < 0.05: *vs* age-matched control, ^#^P < 0.05: *vs* age-matched vehicle-treated counterpart.

Subchondral bone in the control groups was regularly aligned with bone marrow cavities evenly distributed across the entire joint (Fig. 2A “Con + Veh”); only a few osteoclasts could be identified (Fig. 3A “Con + Veh”). Localized subchondral bone loss and enlarged bone marrow cavities were evident in both the 4-week and 8-week saline-injected experimental groups (Fig. 2A “Exp + Veh”). Increases in osteoclast cell numbers were observed in the center and posterior part of the condyles (Fig. 3A “Exp + Veh). In these experimental groups, data analyses revealed significant decreases in BMD and BVF (Fig. 2B), but significant increases in Tb.Sp (Fig. 3B), Oc.N, Oc.S/BS (Fig. 3C), and mRNA levels of cathepsin K (Cask), TRAP, calcitonin receptor (CTR) (Fig. 3D), Runx2, type I collagen (COL1) and osteocalcin (OCN) (Fig. 4C), when compared with the respective age-matched control groups (all p < 0.05).

Condylar subchondral bone loss and osteoclast hyperfunction were suppressed after 4 and 8 weeks of intraperitoneal propranolol treatment in the experimental rats (Fig. 2A “Exp + PRO”). This observation was supported by increased BMD, BVF and Tb.Th (Fig. 2B), as well as decreased Tb.Sp (Fig. 3B), Oc.N, Oc.S/BS (Fig. 3C) and mRNA levels of Cask, TRAP and CTR (Fig. 3D), when compared to the vehicle-treated counterparts (all p < 0.05). However, propranolol treatment had no significant effects on osteocalcin-positive osteoblast number and the mRNA levels of Runx2, COL1 and OCN in the condylar subchondral bone of both the 4-week and 8-week experimental rats (all p > 0.05; Fig. 4A–C “Exp + PRO”). After propranolol treatment, the parameters of trabecular microstructure and osteoclast activities of condylar subchondral bone in the 4-week and 8-week experimental groups were not significantly different from those in the corresponding control groups (p > 0.05, Fig. 2B and Fig. 3B–D).

Isoproterenol treatment further intensified the subchondral bone loss and osteoclast hyperfunction induced by 4 and 8 weeks of abnormal occlusion (Fig. 2 and Fig. 3 “Exp + ISO”). These observations were also supported by decreases in BMD, BVF, Tb.Th, Tb.N (Figs 2B and 3B), and increases in Tb.Sp (Fig. 3B), Oc.N, Oc.S/BS (Fig. 3C) and mRNA levels of Cask, TRAP and CTR (Fig. 3D), when compared to the vehicle-treated counterparts (all p < 0.05). These features were accompanied by a significant decrease in the number of osteoblasts and in the mRNA levels of Runx2, COL1 and OCN only at the 8-week time-point, but not at the 4-week time-point (p < 0.05; Fig. 4A–C).

Four-week and 8-week propranolol treatment and 4-week isoproterenol treatment have no significant effects on the trabecular BMD and microstructure for the control rats (p > 0.05, Supplementary Fig. 1), while 8-week isoproterenol treatment of control rats significantly decreased their BMD and BVF, but increased their Tb.Sp comparing to the vehicle-treated counterparts (p < 0.05, Supplementary Fig. 1).

### Propranolol did not rescue cartilage degradation induced by abnormal occlusion, while isoproterenol aggravated it

The condylar cartilage in the Con + Veh group was well organized, and exhibited rich and even distribution of proteoglycans (Fig. 2A). Cartilage degradation was observed in both the 4-week and 8-week Exp + Veh groups, typically in the form of cartilage thinning and proteoglycans loss, as described previously (Fig. 2A)^17^,^18^,^19^,^20^,^21^,^22^. In these experimental groups, data analyses revealed significant decreases in the thickness of hypertrophic and total layers and the percent area of proteoglycans, when compared to the respective age-matched control groups (Fig. 2B, all p < 0.05). Cartilage thickness and the percent area of proteoglycans in the 4-week and 8-week Exp + PRO groups were not different from those in the vehicle-treated counterparts and were still significantly lower than the Con + Veh groups (Fig. 2B, p < 0.05). In the 4-week and 8-week Exp + ISO groups, cartilage degradation in the form of cartilage thinning and proteoglycans loss was more severe when compared to those in the vehicle-treated counterparts. These morphologic observations were supported by decreased thickness of hypertrophic and total layers and the percent area of proteoglycans (Fig. 2B, p < 0.05).

### Abnormal occlusion induced concomitant increases in expression of Adrb2 by MSCs and RANKL in condylar subchondral bone

**Figure 5:**
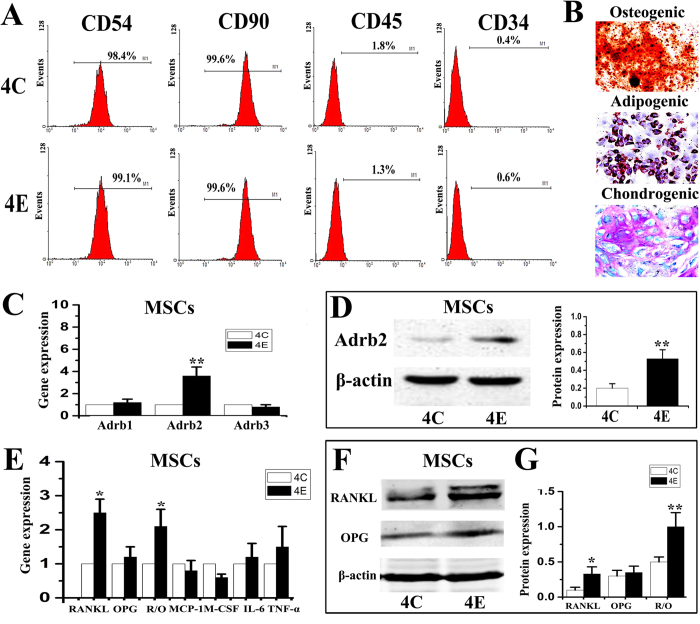
Characterization and multi-lineage differentiation capacity of mesenchymal stem cells (MSCs) isolated from condylar subchondral bone of 4-week control (4C) and 4-week experimental (4E) rats, and comparison of their expression of Adrb1/2/3 and pro-osteoclastic factors. **A**: Flow cytometry analysis showed that MSCs from control and experimental condylar subchondral bone were similarly positive for MSC-associated markers (CD54 and CD90), and negative for hematopoietic markers (CD45 and CD34). **B**: Osteogeneic, adipogenic and chondrogenic differentiation of MSCs isolated from condylar subchondral bone. **C**: Real-time PCR analysis of the mRNA expression of β-adrenergic receptors (β-ARs) in MSCs isolated from condylar subchondral bone of 4-wk control and experimental rats. **D**: Western blot analysis of the Adrb2 expression in MSCs. **E**: Real-time PCR analysis of the mRNA expression of pro-osteoclastic factors in MSCs. **F**: Western blot analysis of the RANKL and OPG expression in MSCs. *P < 0.05, **P < 0.01.

Mesenchymal stem cells isolated from both 4-week control and experimental condylar subchondral bone were positive for rat MSC-associated markers (CD54 and CD90), but failed to express hematopoietic markers (CD45 and CD34) (Fig. 5A). These MSCs possessed multi-lineage differentiation capacities (Fig. 5B). The mRNA (Fig. 5C,E) and protein expression (Fig. 5D,F) of Adrb2, RANKL and RANKL/OPG by MSCs harvested from the 4-week experimental group were significantly higher than those from the 4-week control group (p < 0.05). However, no significant differences in the expression of Adrb1, Adrb3 (Fig. 5C), OPG, MCP-1, M-CSF, IL-6 and TNF-α (Fig. 5E) were detected between the 4-week control and experimental groups (p > 0.05).

### Activation of Adrb2-protein kinase A (PKA) axis in MSCs from condylar subchondral bone increased their expression of RANKL and promoted osteoclastogenesis

**Figure 6:**
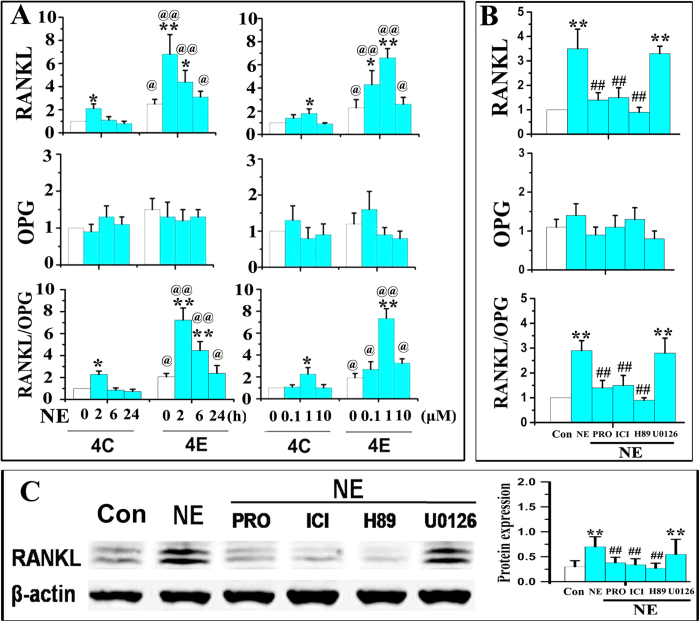
Expression of RANKL, OPG and RANKL/OPG ratio by MSCs after norepinephrine (NE) treatment. **A** and **B**: Real-time PCR of the expression RANKL, OPG and RANKL/OPG ratio by MSCs after NE stimulation. The MSCs were isolated from condylar subchondral bone of 4-week control (4C) and 4-week experimental (4C) rats, and were treated by NE for 2, 6 and 24 h at 0.1, 1 and 10 μM, respectively (**A**). In addition, MSCs from 4-week experimental rats were stimulated by 1 μM NE for 2 h alone, or pre-treated for 1 h with 10 μM propranolol (PRO), 1 μM ICI 118,551 (ICI), 5 μM H-89 or 5 μM U-0126 (U0126) (**B**). *P < 0.05: *vs* vehicle-treated MSCs, **P < 0.01: *vs* vehicle-treated MSCs; ^@^P < 0.05: *vs* the corresponding NE-treated MSCs from 4-week control (4C), ^@@^P < 0.01: *vs* the corresponding NE-treated MSCs from 4-week control (4C), ^##^P < 0.01: *vs* NE-treated MSCs. **C**: Western blot of the RANKL expression by MSCs stimulated by 1 μM NE for 2 h alone, or pre-treated for 1 h with 10 μM PRO, 1 μM ICI, 5 μM H-89 or 5 μM U0126. The MSCs were isolated from4-week experimental rats.

In MSCs isolated from condylar subchondral bone of 4-week control and experimental rats, mRNA expression of RANKL and RANKL/OPG were both maximally increased after 2 h of norepinephrine stimulation that gradually decreased with the time of stimulation (p < 0.05, Fig. 6A). There were no significant difference in the mRNA expressions of RANKL and RANL/OPG between the 24 h NE stimulation group and the time-matched control (p > 0.05, Fig. 6A). Up-regulations of RANKL and RANKL/OPG by norepinephrine stimulation were significantly increased when norepinephrine was administered at the concentration of 1 μM; there was an increasing tendency of up-regulation when the concentration of norepinephrine increased from 0.1 to 1 μM, but a decreasing tendency of up-regulation when the concentration of norepinephrine further increased from 1 to 10 μM (p < 0.05, Fig. 6A). For all time-periods and norepinephrine concentrations, expression of RANKL and RANKL/OPG were all significantly higher in MSCs harvested from the 4-week experimental group, when compared to the corresponding 4-week control group (p < 0.05, Fig. 6A).

Both propranolol (non-selective β-receptor antagonist) and ICI 118,551 (selective β2-receptor antagonist) blocked norepinephrine-induced increased expression of RANKL at the gene (Fig. 6B) and protein levels (Fig. 6C). There was no significant difference between the blocking effects of these two β-receptor antagonists (p > 0.05). The selective PKA inhibitor H-89 also suppressed the norepinephrine-induced increases in RANKL mRNA and protein expression (p < 0.05, Fig. 6B,C). However, the ERK1/2 inhibitor U-0126 had no effect on RANKL expression (p > 0.05, Fig. 6B,C).

**Figure 7:**
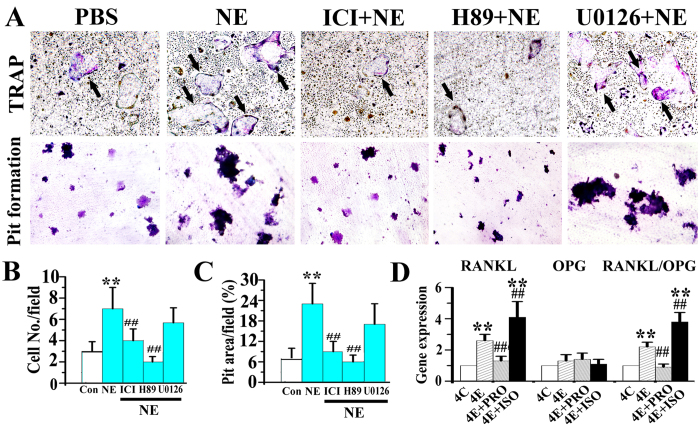
The pro-osteoclastic activity of norepinephrine (NE)-stimulated MSCs. The MSCs from 4-week experimental rats were stimulated with 1 μM NE for 2 h alone, or pre-treated for 1 h with 1 μM ICI 118,551 (ICI), 5 μM H-89 or 5 μM U-0126 (U0126) and then stimulated by NE. These MSCs were co-cultured with bone marrow macrophages. **A**: Representative images of TRAP-stained osteoclasts (upper row) and resorption pits (lower row) produced by the bone marrow marcrophages. **B and C**: Barcharts depicting the number of TRAP-positive multinucleated cells (10 fields) and area of resorption pits. **D**: Real-time PCR of the expression RANKL, OPG and RANKL/OPG ratio by MSCs isolated from the condylar subchondral bone of 4-week control rats (Con) and experimental rats (Exp) treated with physiological saline (Veh), propranolol (PRO) or isoproterenol (ISO).

Norepinephrine-treated MSCs stimulated the formation of TRAP-positive multinuclear osteoclasts from BMMs and increase the percentage area of dentin resorption pits produced by the transformed osteoclasts (all p < 0.05, Fig. 7A–C). These effects were completely blocked by ICI 118,551 and H89 (both p < 0.05), but were not altered by U-0126 (p > 0.05, Fig. 7A–C). Notably, when compared to vehicle-treated 4-week experimental rats (“4E” in Fig. 7D), mRNA expression of RANKL and RANKL/OPG by MSCs of 4-week experimental rats injected with propranolol (“4E + PRO” in Fig. 7D) were significantly decreased, while those in 4-week experimental rats injected with isoproterenol (“4E + ISO” in Fig. 7D) were significantly increased (p < 0.05).

## Discussion

Increased subchondral bone remodeling and sprouting of sympathetic nerve fibers have been identified from the osteochondral junction of osteoarthritic joints^31^,^32^. Nevertheless, the role of sympathetic nerves in the regulation of abnormal subchondral bone remodeling in OA remains obscure. Using a rat TMJ OA model, the present study provides novel insights to this issue by demonstrating that the sympathetic tone is activated in the condylar subchondral bone by iatrogenically-created abnormal occlusion. This is evidenced by increased level of bone norepinephrine, increased sprouting of tyrosine hydroxylase-positive sympathetic nerve fibers, and increased expression of β2-adrenergic receptors (Adrb2) at the gene and protein levels in the condylar subchondral bone. Activation of sympathetic tone is accompanied by progressive subchondral bone loss and increased osteoclastic activities. These observations are further supported by showing that the use of propranolol, a β-antagonist, inhibits subchondral bone loss and suppresses osteoclast activities in the condyles of the experimental rats, while the use of isoproterenol, a β-agonist exacerbates the aforementioned responses. In addition, MSCs isolated from the subchondral bone of experimental rats exhibiting TMJ OA-like changes express increased levels of Adrb2 and RANKL. This observation is corroborated by the results generated from a separate set of experiments, wherein the application of norepinephrine to activate Adrb2 receptors in MSCs derived from non-OA (control rats) and OA TMJs (experimental rats) promotes osteoclast differentiation by increasing the RANKL/OPG ratio to different extents. Taken together, these results validate the hypothesis that abnormal occlusion induced activation of the sympathetic tone elicits osteoclast-mediated subchondral bone remodeling in a rat TMJ OA model.

Subchondral bone undergoes constant remodeling in response to joint loading. Activation of TGF–β1^46^ and Wnt signals^47^ in the subchondral bone in response to altered mechanical loading has been reported to be associated with pathological OA changes in rodent models. Nevertheless, the manner in which bone sanities changes in the loading environment remains obscure. Previous studies have shown that the sympathetic tone plays regulatory roles in the bone mechanoadaptive response through the release of norepinephrine^25^,^26^,^27^,^48^. The present study provides further evidence that aberrant mechanical loading on the rat TMJ precipitates subchrondral bone loss that is concomitant with increased sympathetic nerve sprouting and norepinephrine levels in the subchondral bone.

Although pharmacological suppression of sympathetic nerve activities by propranolol alleviated condylar subchondral bone loss in experimental rats, the use of propranolol alone did not significantly induce subchondral bone growth in control rats (Supplemental Fig. 1). These results support the notion that β-blocking interferes with the mechanism of abnormal occlusion induced osteoclastic bone resorption, instead of merely enhancing bone anabolism. Emerging clinical evidence indicates that blockage of β-adrenoreceptor signal via propranolol is an effective pain-relieving modality for patients suffering from temporomandibular disorders^49^,^50^. Norepinephrine released at the site of TMJ contributes to the development of hyperalgesic joint pain through activation of β2-adrenoceptors in a carrageenan-induced murine arthritis model^51^. Because pain in arthritic joints may originate from subchondral bone^32^, the association between the peripheral analgesia mechanism of propranolol and its protective effect on subchondral bone destruction requires further investigation.

It has been reported that administration of 20 μg/g/day doses of propranolol 5 days per week for 10 weeks had a negative effect on heart functions but no significant protective effects on bone mass in ovariectomized (OVX) rats, while 0.1 μg/g of propranolol prevented OVX-induced bone loss without any harmful effects on heart hemodynamic parameters^52^. In the present study, 20 μg/g of propranolol was administered intraperitoneally to the rats twice a week for 4 or 8 weeks, which inhibited condylar subchondral bone loss induced by abnormal dental occlusion. This result is in accordance with previous studies reporting that the same dose of propranolol could effectively prevent hindlimb bone loss induced by tail suspension^26^ and alveolar bone loss induced by orthodontic tooth movement^28^. In addition, we analyzed condylar subchondral bone in control rats treated by 20 μg/g propranolol or vehicle as a reference. There were no significant differences in BMD and trabecular microstructures between the two groups at 4-week and 8-week time-points (Supplementary Fig. 1). This suggested that 20 μg/g propranolol used in the present study did not cause systematic effects on condylar subchondral bone. However, we cannot totally exclude the possibility that propranolol used in our experiments may have affected bone mass via affecting heart functions, and these issues have to be elucidated in the future. Furthermore, consecutive injection (5 days per week) of low dose (0.1 μg/g) of propranolol would be tested to evaluate their bone sparing effects in the present animal model.

β2-AR is expressed by MSCs, osteoblasts, osteoclast precursors and osteoclasts^24^. Stimulation of β2-AR inhibits proliferation and osteogenic differentiation of osteoblasts^24^, and promotes the differentiation and maturation of osteoclast precursors *in vitro* and *in vivo*^24^,^53^,^54^. In addition, activation of β2-AR in osteoblasts up-regulates RANKL expression to indirectly promote osteoclast differentiation and function^24^. Because no significant effect of norepinephrine or isoproterernol on osteoblast growth, differentiation or function *in vitro* was previously reported^54^, and the present propranolol treatment and 4-week isoproterernol treatment of experimental rats primarily suppressed osteoclast hyperfunction in the subchondral bone, but has negligible effect on osteocalcin-positive osteoblast number and the mRNA levels of Runx2, COL1 and OCN, the present study focused on the pro-osteoclastic effect of β2-AR activation in MSCs. Furthermore, recent studies have shown that MSCs isolated from the destructive bone microenviroment promote bone resorption by osteoclasts^34^,^35^, and the activities of these MSCs are regulated by signals derived from adrenergic nerve fibers of the sympathetic nervous system^36^,^37^. Hence, the expression of Adrb in MSCs isolated from condylar subchondral bone was investigated in the present work, to identify their regulatory roles on osteoclast development. Nevertheless, the present data is limited to demonstrating the primary etiology of abnormal subchondral bone remodeling in TMJ OA. Mice with conditional knockout expression of Adrb2 from MSCs (Nestin-Cre)^37^, osteoblasts (osteocalcin-Cre)^55^ or osteoclasts (TRAP-Cre)^56^, through Adrb2 Flox/Flox mice mating with specific Cre-transgenic mice, are needed to clarify which cell type (MSC, osteoblast or osteoclast) is responsible for abnormal bone remodeling of TMJ OA.

The observations that Adrb2 expression increased in MSCs isolated from rats with subchondral bone loss, while those of Adrb1 and Adrb3 were unaltered, highlight the important role played by Adrb2 in sympathetic tone-regulated bone remodeling^24^. Changes in RANKL expression by those MSCs in response to propranolol and isoproterenol treatment suggest that MSCs enhance osteoclastic activity through activation of Adrb2 signals by the sympathetic tone. This speculation was confirmed by additional experiments showing that norepinephrine intensified the expression of RANKL by MSCs isolated from the subchondral bone of both control and experimental rats, and selective β2-receptor antagonist ICI 118,551 suppressed RANKL expression by norepinephrine stimulated MSCs.

**Figure 8:**
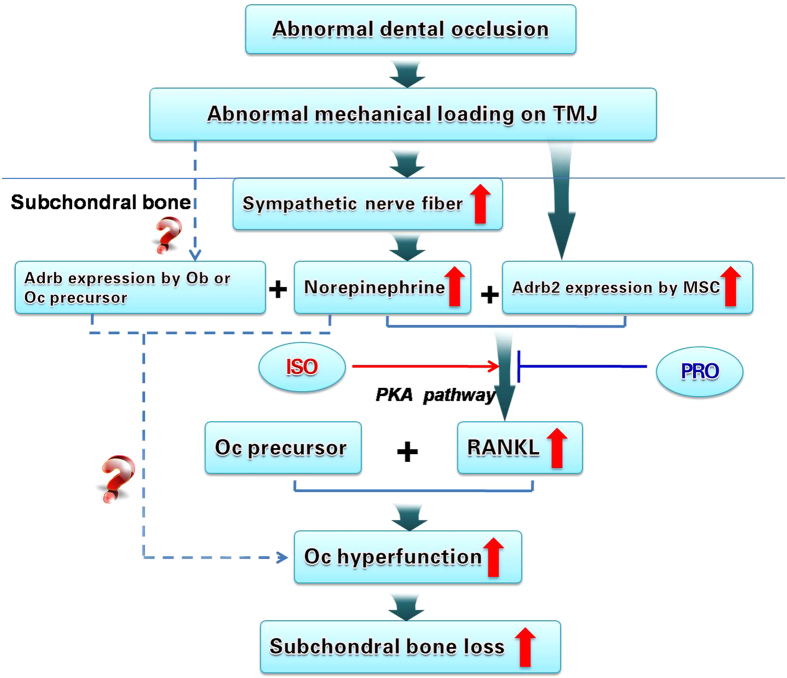
Schematic model of elevated β2-AR (Adrb2) signal transduction in subchondral bone loss of TMJOA. In condylar subchondral bone, increased sprouting of sympathetic nerve fibers and norepinephrine (NE) level, and increased expression of Adrb2 by MSCs occurs in response to abnormal mechanical loading. The accumulated high concentration of NE, via PAK pathway, promotes the MSCs to secret increased amount of RANKL, which stimulate the formation mature osteoclast and subsequent subchondral bone loss for TMJOA progression. In addition, activation of Adrb signal in osteoblast (Ob) or osteoclast (Oc) might also play a role in the subchondral bone loss of TMJOA.

Activation of cell surface β2-AR by the sympathetic tone stimulates intracellular signal transduction via the cyclic adenosine monophosphate (cAMP)/protein kinase A (PKA) cascade, which, in turn, induces gene expression in the nucleus^24^. Extracellular signal-regulated kinase (ERK) 1/2 pathway has also been implicated in epinephrine-induced β2-AR signal transduction^57^. Hence, the potential contribution of the PKA and ERK1/2 pathways on increased RANKL expression by MSCs via β2-AR activation was further investigated. Inhibition of the PKA pathway, but not the ERK1/2 pathway, blocked the expression of RANKL by norepinephrine-stimulated MSCs and abolished their pro-osteoclastic effects. These results attest that under the stimulation by the sympathetic tone, the β2AR-PKA axis is responsible for mediating the osteoclast stimulating potential of MSCs in condylar subchrondral bone (Fig. 8).

In addition, β2-AR expression has also been detected in knee articular chondrocytes^58^, growth plate chondrocytes^59^ and condylar chondrocytes, similar to the features depicted in Fig. 1C. It has been reported that regulation of chondrocytes through their expression of β2-AR stimulates their growth and inhibits their differentiation^60^. The present results showed that 8-wk isoproterenol administration further aggravated cartilage degradation induced by abnormal occlusion, as demonstrated by intensive subchondral bone loss and osteoclast hyperfunction. These observations were in accordance with recent findings that the loss of subchondral bone triggered degradation of the overlying cartilage through aggravation of the biomechanical environment. Although results from these studies provided the rationale for using bone antiresorptives and anabolics as potential treatment modalities for OA, the present propranolol treatment did not effectively rescue cartilage degradation induced by abnormal occlusion. Thus, other pathogenic mechanisms maybe involved in the development of cartilage degradation, apart from subchondral bone remodeling induced by norepinephrine. Furthermore, intraperitoneal injection used in the present study could not distinguish the targeted effects of agonist from antagonist on chondrocytes or bone cells. Further studies by intra-articular injection will be helpful in elucidating the direct effect of β2-AR signal on condylar cartilage metabolism in subjects with normal condylar conditions or OA.

In summary, the present study demonstrates that abnormal occlusal loading induces subchondral bone loss in the mandibular condyles through activation of β2-adrenergic receptors by the sympathetic tone. Increased RANKL secretion by condylar MSCs in response to this signal transduction process, in turn, exacerbates osteoclastic activity (Fig. 8).

## Additional Information

**How to cite this article**: Jiao, K. *et al.* β2-adrenergic signal transduction plays a detrimental role in subchondral bone loss of temporomandibular joint in osteoarthritis. *Sci. Rep.* **5**, 12593; doi: 10.1038/srep12593 (2015).

## Supplementary Material

Supplementary Information
