# Supplementary material for: β2-adrenergic signal transduction plays a detrimental role in subchondral bone loss of temporomandibular joint in osteoarthritis
Source: Sci Rep. 2015 Jul 29;5:12593. doi: 10.1038/srep12593 (PMC4518212; doi:10.1038/srep12593)
Supplement: Supplementary Information [file srep12593-s1.doc]

**Supplementary Information**

**β2-adrenergic signal transduction plays a detrimental role in subchondral bone loss of temporomandibular joint in osteoarthritis**

Kai Jiao^1,#^, Li-Na Niu^2,#^, Qi-hong Li^3#^, Gao-tong Ren^4^, Chang-ming Zhao^4^, Yun-dong Liu^1^, Franklin R. Tay^5,*^, Mei-qing Wang^1,*^

**
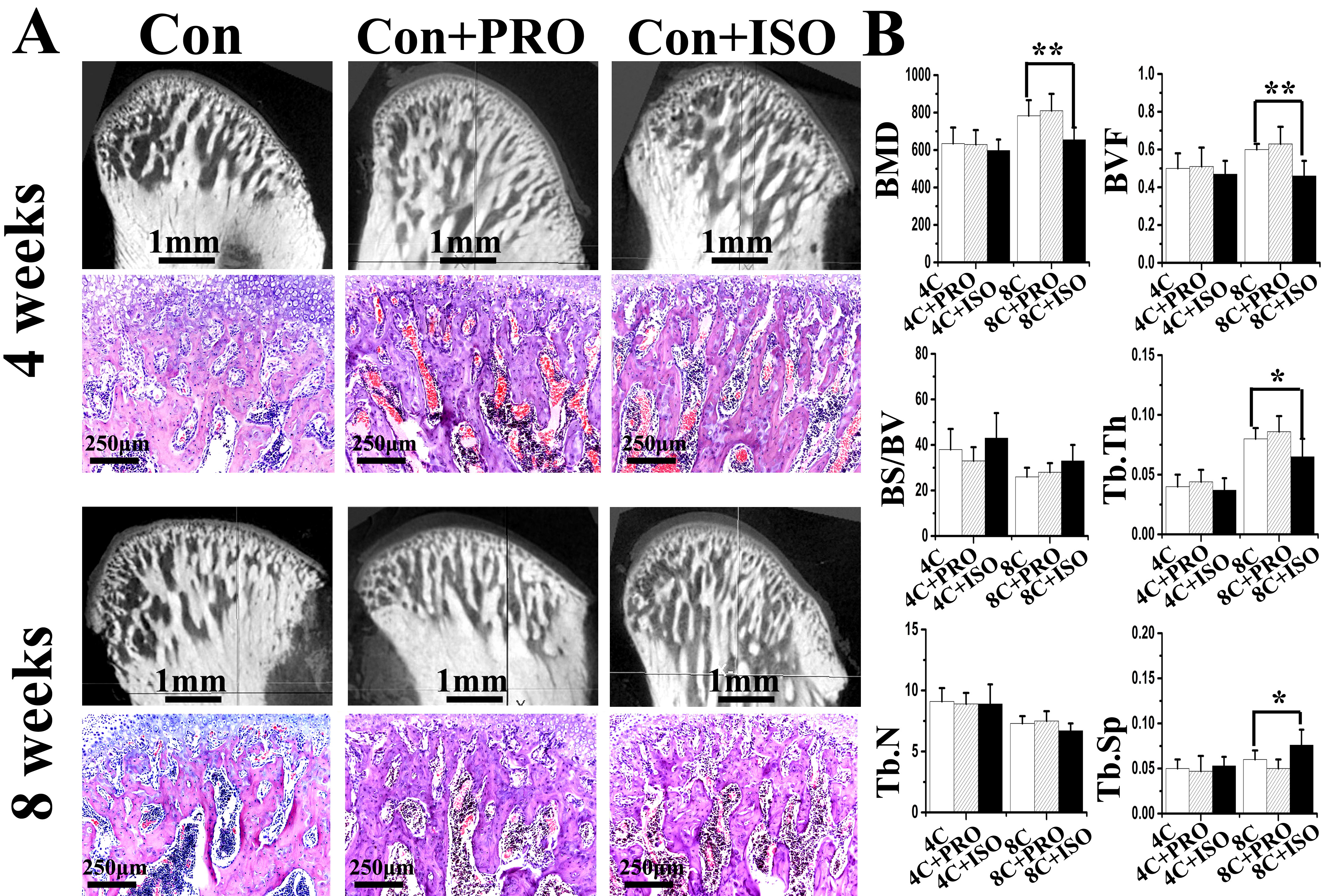
Supplemental Figure.1** Condylar subchondral bone changes of 4-week and 8-week control rats (Con) treated with physiological saline (Veh), non-selective β-blocker propranolol (20 μg/g, PRO), or β-adrenergic receptors agonist isoproterenol (5 μg/g, ISO). **A**: Representative images of micro-computed tomography and H&E staining of the mandibular condyle. **B**: Analysis of bone mineral density (BMD) and different parameters representing trabecular microstructures of the condylar subchondral bone based on the reconstructed micro-computed tomography images.

**Supplemental Table I.** Gene primers

| **genes** | **forward primer** | **reverse primer** |
| --- | --- | --- |
| Adrb1 | TCTGTGAGCTCTGGACTTCGGTA | GATGACACACAGGGTCTCGATG |
| Adrb2 | GATTGCAGTGGATCGCTATGTTG | GACCACTCGGGCCTTATTCTTG |
| Adrb3 | ccctttcttcctactgctttcct | tttgtgcctattgtgagagatggt |
| RANKL | TCGGGTTCCCATAAAGTCAG | CTTGGGATTTTGATGCTGGT |
| OPG | TGGGAATGAAGATCCTCCAG | GAGGAAGGAAAGGGCCTATG |
| MCP-1 | CTATGCAGGTCTCTGTCACGCTTC | CAGCCGACTCATTGGGATCA |
| M-CSF | CATCCAGGCAGAGACTGACA | TTCGCGCAGTGTAGATGAAC |
| IL-6 | CCACTTCACAAGTCGGAGGCTTA | GTGCATCATCGCTGTTCATACAATC |
| TNF-α | AACTCGAGTGACAAGCCCGTAG | GTACCACCAGTTGGTTGTCTTTGA |
| GAPDH | TGTGTCCGTCGTGGATCTGA | TTGCTGTTGAAGTCGCAGGAG |
